# Circulating cell-free DNA as an integrative biomarker in breast cancer: correlation with molecular subtypes, mutation status, and treatment response

**DOI:** 10.3389/fonc.2026.1737210

**Published:** 2026-02-18

**Authors:** Ranjana Solanki, Ashmeet Kaur, Ravindra Gothwal, Kamal Kishore Lakhera, Bhuvanesh Sukhlal Kalal, Urvashi Vijay

**Affiliations:** 1 Department of Pathology, SMS Medical College, Jaipur, Rajasthan, India; 2 Department of Radiation Oncology, SMS Medical College, Jaipur, Rajasthan, India; 3 Department of Surgical Oncology, SMS Medical College, Jaipur, Rajasthan, India; 4 Multidisciplinary Research Unit, SMS Medical College, Jaipur, Rajasthan, India

**Keywords:** breast cancer, cell-free DNA, cfDNA dynamics, liquid biopsy, molecular subtypes, PIK3CA mutation, precision oncology, TP53 mutation

## Abstract

**Background:**

Cell-free DNA (cfDNA) is an emerging non-invasive biomarker in oncology. While its diagnostic and prognostic roles in breast cancer are established, limited studies have examined cfDNA dynamics in relation to molecular subtypes and mutation profiles, particularly in underrepresented populations.

**Objectives:**

To assess the diagnostic and predictive utility of cfDNA in breast cancer by analyzing its association with molecular subtypes, TP53 and PIK3CA mutations, and treatment response across dual timepoints.

**Methods:**

Fifty histologically confirmed breast cancer patients and 20 age-matched healthy controls were enrolled. Baseline and follow-up cfDNA levels were quantified in 32 paired cases. cfDNA dynamics (ΔcfDNA) were analyzed by subtype and mutation status. Diagnostic and predictive performance was evaluated using ROC (Receiver operating characteristic) and Precision–Recall (PR) curve analyses. Mutation distribution and subtype frequencies were compared with global and regional datasets.

**Results:**

Baseline cfDNA levels were significantly elevated in breast cancer patients compared to controls (p < 0.001), with highest levels in TNBC (Triple-negative breast cancer) and HER2-enriched subtypes and lowest in Luminal A. Follow-up cfDNA remained persistently elevated in aggressive subtypes and TP53-mutated cases, suggesting molecular residual disease. ΔcfDNA trends varied, with TP53-mutated TNBC showing the highest increases. PIK3CA mutations were observed in 36% of cases, predominantly E545K (28%), while H1047R was rare (2%). TP53 mutations were detected in 10%. PR analysis showed moderate predictive value for TP53 (Average Precision = 0.72) and PIK3CA (Average Precision = 0.71). A cfDNA threshold of 137 ng/mL achieved 91% sensitivity and 92% specificity (Area Under the Curve = 0.97).

**Conclusion:**

cfDNA levels reflect tumor burden, subtype biology, and mutation status, offering real-time, non-invasive insight into disease dynamics. The high E545K and rare H1047R frequencies suggest underexplored, population-specific genomic patterns. cfDNA trends across subtypes range from persistent elevation in TNBC to decline in Luminal A, suggesting its potential role in treatment monitoring. These findings underscore cfDNA’s relevance to evolving precision oncology frameworks, particularly in biopsy-limited and resource-constrained settings.

## Graphical Abstract

**Figure:**
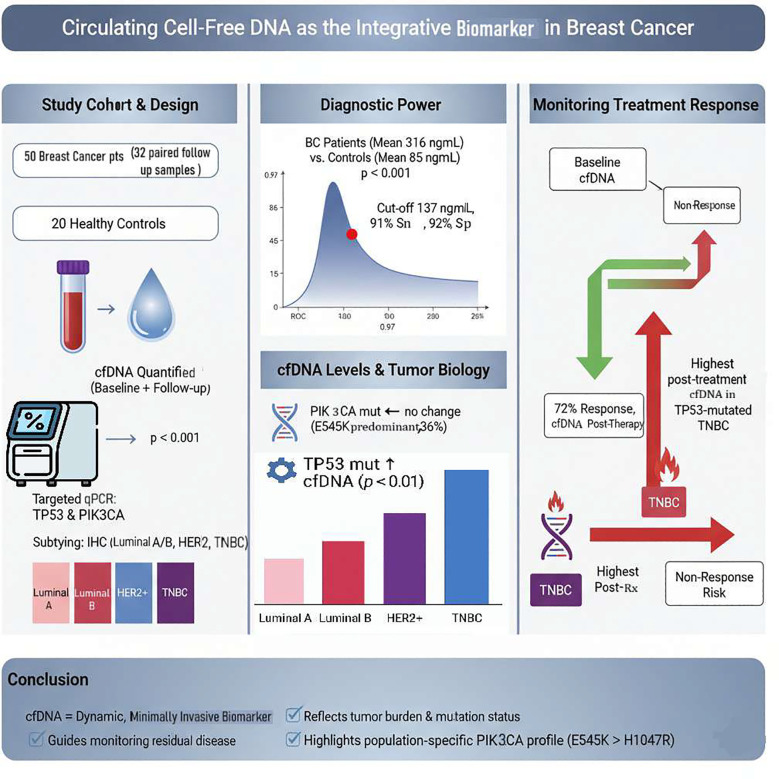
Graphical summary illustrating the study workflow and key findings, highlighting cfDNA dynamics across breast cancer molecular subtypes, mutation status (TP53 and PIK3CA), and treatment response.

## 1 Introduction

Breast cancer is the most commonly diagnosed cancer and the leading cause of cancer-related mortality in women worldwide, with 2.3 million new cases reported in GLOBOCAN 2022. In India, it accounts for 28.2% of all female cancers, with urban areas showing the highest incidence rates (1–3). This rising burden is driven by lifestyle changes, limited screening, delayed diagnosis, and disparities in healthcare (4).

Conventional methods for detecting and profiling breast tumors, such as imaging and tissue biopsies, form the cornerstone of clinical diagnostics, yet they are not without drawbacks. Mammography, the primary screening tool, often shows limited sensitivity and specificity, especially in dense breast tissue (5). Biopsies, though the gold standard, are invasive and impractical for repeated assessments. Single biopsies may miss tumor heterogeneity, especially in metastatic cases. These limitations restrict the ability of conventional methods to track disease progression or response to therapy.

To address these challenges, liquid biopsy has emerged as a transformative non-invasive diagnostic modality. Liquid biopsy, particularly cfDNA analysis, enables real-time tumor monitoring through a simple blood sample, reflecting tumor burden and mutation status (4, 6). Other liquid biopsy approaches include circulating tumor cells (CTCs), exosomal RNA, and methylated DNA or RNA markers. While CTCs provide cellular-level information, they are technically challenging to isolate. Methylated RNA and DNA markers offer high specificity but often require complex workflows and are still evolving in clinical translation. cfDNA offers a balance of simplicity, sensitivity, and scalability, making it suitable for integration into routine practice. However, its utility in mutation detection may be limited by low allele frequency or fragmented DNA.

TP53 (30–35%) and PIK3CA mutations are key cfDNA targets in breast cancer, linked with aggressiveness and therapeutic response (7, 8). Studies, including Indian cohorts, validate the detection of these mutations via qPCR (quantitative polymerase chain reaction) and next-generation sequencing (9–12). While NGS enables broader mutation detection, PCR-based assays offer faster, cost-effective detection of hotspot mutations, making them suitable for routine clinical settings. Dynamic cfDNA changes during treatment can indicate response or resistance earlier than imaging (13). This ability underscores the value of liquid biopsy as a dynamic tool for ongoing surveillance and informed treatment adaptation throughout the disease course (14).

Despite global validation, cfDNA diagnostics remain underutilized, particularly in low- and middle-income countries. Our study not only addresses gaps in regional practice but also contributes to global efforts to diversify genomic data, enabling broader applicability of cfDNA biomarkers in heterogeneous populations and resource-limited settings (4, 15). Whole exome sequencing-based studies have recently reported a high prevalence of TP53 and PIK3CA mutations among Indian breast cancer patients, revealing unique mutational patterns with potential clinical relevance (11).

## 2 Materials and methods

### 2.1 Study design and participants

This case-control study was conducted at Sawai Man Singh Medical College (SMS), Jaipur, Rajasthan, India, with participants recruited from the departments of Surgical Oncology, Radiation Oncology, and Pathology. Ethical approval was obtained from the Institutional Review Board, Office of the Ethics Committee, S.M.S. Medical College and Attached Hospitals, Jaipur (No.: 4012/MC/EC/2018), and informed consent was acquired from all participants. The study was carried out from September 2020 to March 2023, funded by the DHR-Multidisciplinary Research Unit (MDRU), SMS Medical College, Jaipur.

**Figure 1:**
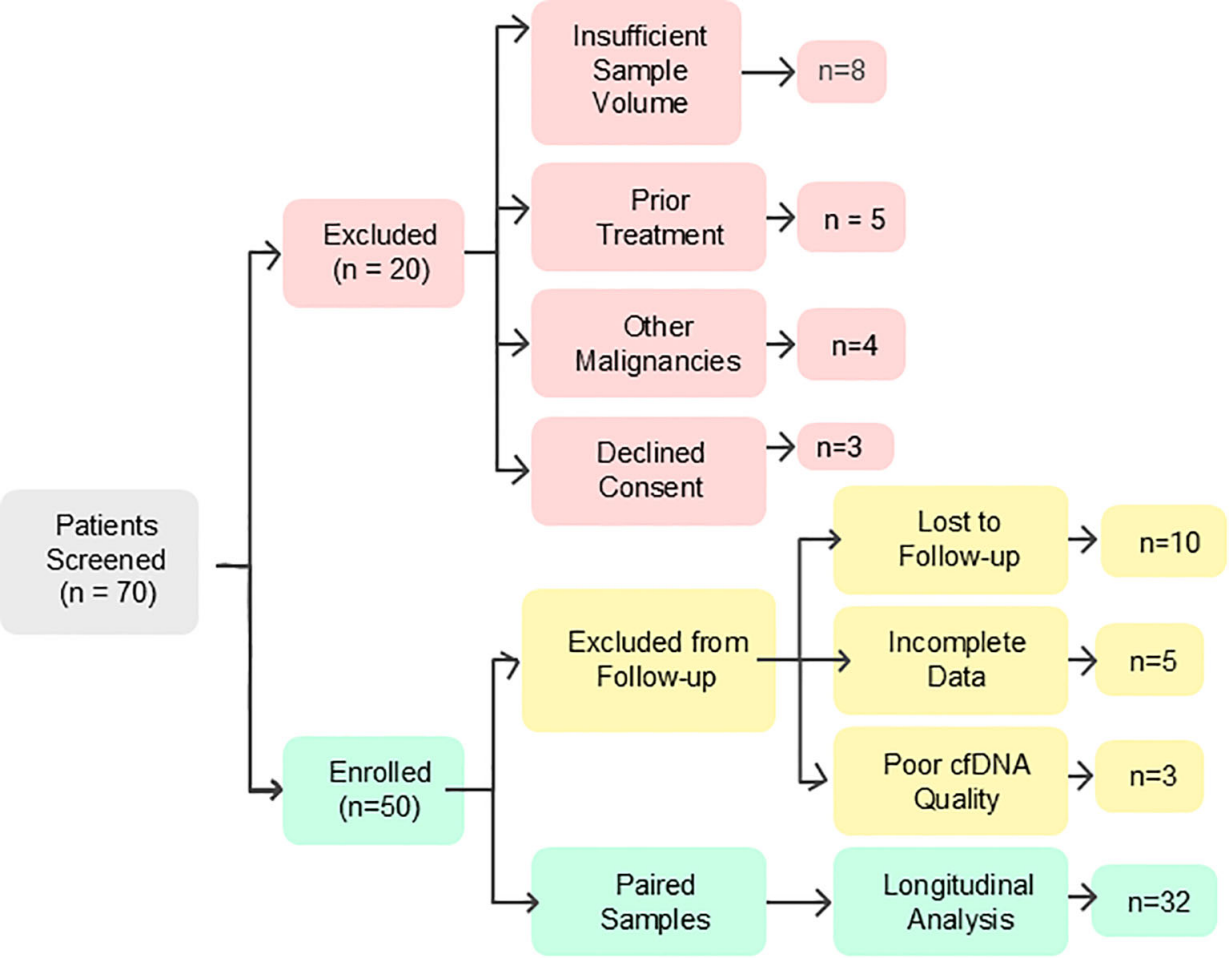
CONSORT-style flow diagram depicting screening, exclusions, and final inclusion of the breast cancer patients with paired cfDNA samples in the longitudinal analysis.

A total of 70 patients were initially screened between September 2020 and December 2020. Twenty patients were excluded due to various reasons including insufficient sample volume (n = 8), prior chemotherapy or radiotherapy (n = 5), history of other malignancies (n = 4), or declined consent (n = 3). Fifty eligible patients were enrolled and provided baseline cfDNA samples between January 2021 and June 2022. Follow-up samples were collected between July 2021 and March 2023, with a median interval of approximately 6 months from baseline (range: 3–9 months), depending on individual treatment schedules and response evaluation. Among these, 18 were excluded from follow-up analysis due to loss to follow-up (n = 10), incomplete clinical data (n = 5), or poor cfDNA quality (n = 3). The final cohort included 32 patients with paired baseline and post-treatment cfDNA samples, analyzed longitudinally between April and May 2023. A participant flowchart (**Figure 1**) summarizes these stages.

### 2.2 Clinical assessment

A total of 50 histologically confirmed breast cancer patients and 20 age- and sex-matched healthy female controls were included in the study, with 32 patients providing paired samples (baseline and follow-up). Patients with a history of other malignancies, prior chemotherapy or radiotherapy, or insufficient sample volume were excluded. Clinical and pathological data, including ER, PR, and HER2 status, were collected and recorded. Hormone receptor testing was performed on FFPE blocks using IHC according to ASCO-CAP 2018 guidelines (16).

Patients received standard-of-care treatment based on tumor subtype and clinical stage. Treatment regimens were recorded but varied according to institutional protocols. As this study focused on cfDNA kinetics across molecular subtypes, detailed treatment stratification was not included in the current analysis.

### 2.3 Sample collection and processing

Peripheral venous blood (5 mL) was collected from each participant into EDTA tubes under aseptic conditions. Samples were processed within 2 hours of collection to minimize leukocyte lysis. Plasma separation was performed by centrifugation at 3000 rpm for 10 minutes at room temperature. The supernatant plasma was carefully aliquoted without disturbing the buffy coat and stored at −80°C until cfDNA extraction.

### 2.4 *cfDNA extraction* and quality assessment

Cell-free DNA was extracted from 500 µL of plasma using the MagMAX™ Cell-Free DNA Isolation Kit (Applied Biosystems, Thermo Fisher Scientific) according to the manufacturer’s bead-based protocol, involving magnetic binding, washing, and elution steps. Extracted cfDNA was eluted in nuclease-free buffer and stored at −80°C until further analysis.

Quantification of cfDNA was performed using a Qubit™ 4.0 Fluorometer (Thermo Fisher Scientific) with the dsDNA High Sensitivity Assay for accurate concentration measurement. cfDNA purity was assessed using Nanodrop spectrophotometry by measuring the A260/280 ratio. Fragment size distribution and integrity were evaluated by 1.5% agarose gel electrophoresis, confirming the expected cfDNA fragment size range of approximately 150–200 bp and excluding gross degradation.

### 2.5 Mutation detection by real-time PCR

Somatic mutations in TP53 and PIK3CA were detected using commercially available TaqMan^®^ Mutation Detection Assays based on castPCR™ (competitive allele-specific TaqMan PCR) technology (Applied Biosystems, Thermo Fisher Scientific). These assays employ allele-specific primers and dual-labeled hydrolysis probes designed for high-sensitivity detection of low-frequency variants in cfDNA.

TP53 mutation screening targeted commonly mutated exonic regions, while PIK3CA analysis focused on established hotspot mutations in the helical and kinase domains. Due to the proprietary nature of the commercial assays, individual primer and probe sequences were not disclosed by the manufacturer. However, all assays were used strictly according to validated manufacturer protocols.

PCR reactions were performed on the Bio-Rad CFX96™ Real-Time PCR Detection System. Each run included appropriate mutant and wild-type controls supplied with the assay kits, as well as no-template controls to monitor contamination. Amplification data were analyzed using system software, and mutation calls were made based on manufacturer-defined thresholds.

A detailed list of targeted genes, mutation hotspots, and corresponding exonic regions is provided in **Supplementary Table S1**.

### 2.6 Statistical analysis

Continuous variables were summarized as mean ± standard deviation (SD) or median with interquartile range, as appropriate. Categorical variables were expressed as frequencies and percentages. Group comparisons were performed using Student’s t-test or ANOVA for normally distributed data, and Mann–Whitney U or Kruskal–Wallis tests for non-parametric data. Categorical variables were compared using Chi-square or Fisher’s exact tests.

Correlations were assessed using Pearson’s or Spearman’s correlation coefficients. Effect sizes were calculated using Cliff’s Delta and Pearson’s r where appropriate. Diagnostic performance of cfDNA was evaluated using Receiver Operating Characteristic (ROC) and Precision–Recall curve analyses, with optimal cut-off values determined using the Youden index. A two-sided p-value <0.05 was considered statistically significant.

## 3 Results

### 3.1 Study population characteristics

This study included a total of 50 histologically confirmed breast cancer patients with a mean age of 48.1 ± 11.8 years (median: 43.0 years; IQR: 40.0–57.0 years). All participants were female. Tumor size distribution revealed 3 patients (6.1%) with tumors ≤2 cm, 31 patients (63.3%) with tumors >2 to <5 cm, and 15 patients (30.6%) with tumors ≥5 cm. A detailed summary of the baseline clinical and molecular characteristics of the study population is provided in **Table 1**. The cohort included a diverse representation of molecular subtypes and tumor burdens, with nearly half classified as TNBC and a high proportion exhibiting high Ki-67 index values.

**Table 1:** Clinical and molecular characteristics of the breast cancer study cohort (n = 50).

Characteristic	Value
Age (years)	**Mean: 48.1 ± 11.8**
	**Median (IQR): 43.0 (40.0–57.0)**
Tumor Size	**≤2 cm: 3 (6.0%)**
	**2–5 cm: 32 (64.0%)**
	**>5 cm: 15 (30.0%)**
Lymph Node Status	**<5 nodes: 32 (64.0%)**
	**≥5 nodes: 18 (36.0%)**
ER Status	**Positive: 20 (40.0%)**
	**Negative: 30 (60.0%)**
PR Status	**Positive: 22 (44.0%)**
	**Negative: 28 (56.0%)**
HER2 Status	**Positive: 7 (14.0%)**
	**Negative: 43 (86.0%)**
Molecular Subtype	**Luminal A: 8 (16.0%)**
	**Luminal B: 14 (28.0%)**
	**HER2-enriched: 5 (10.0%)**
	**TNBC: 23 (46.0%)**
Ki-67 Index	**Low (≤14%): 9 (18.0%)**
	**Intermediate (15–20%): 19 (38.0%)**
	**High (>20%): 22 (44.0%)**
TP53 Mutation	**Mutated: 5 (10.0%)**
	**Wild-type: 45 (90.0%)**
PIK3CA Mutation	**Mutated: 18 (36.0%)**
	**Wild-type: 32 (64.0%)**

Immunohistochemistry (IHC) profiling showed that ER and PR were positive in 22 patients (44%) and negative in 28 patients (56%). HER2 overexpression was observed in 7 patients (14%), while 43 patients (86%) were negative. Molecular subtypes were categorized as follows: Luminal A (8 patients, 16%), Luminal B (14 patients, 28%), HER2-enriched (5 patients, 10%), and Triple Negative Breast Cancer (TNBC) (23 patients, 46%).

Among the 50 patients, 9 (18.0%) had a low Ki-67 index (≤14%), 19 (38.0%) had an intermediate index (15–20%), and 22 (44.0%) had a high index (>20%). TP53 mutation was detected in 5 cases (10%), while PIK3CA mutations were found in 18 cases (36%), including E545K in 14 cases (28%), E542K in 3 cases (6%), and H1047K in 1 case (2%). The remaining patients exhibited wild-type profiles: 45 cases (90%) for TP53 and 32 cases (64%) for PIK3CA.

**Figure 2:**
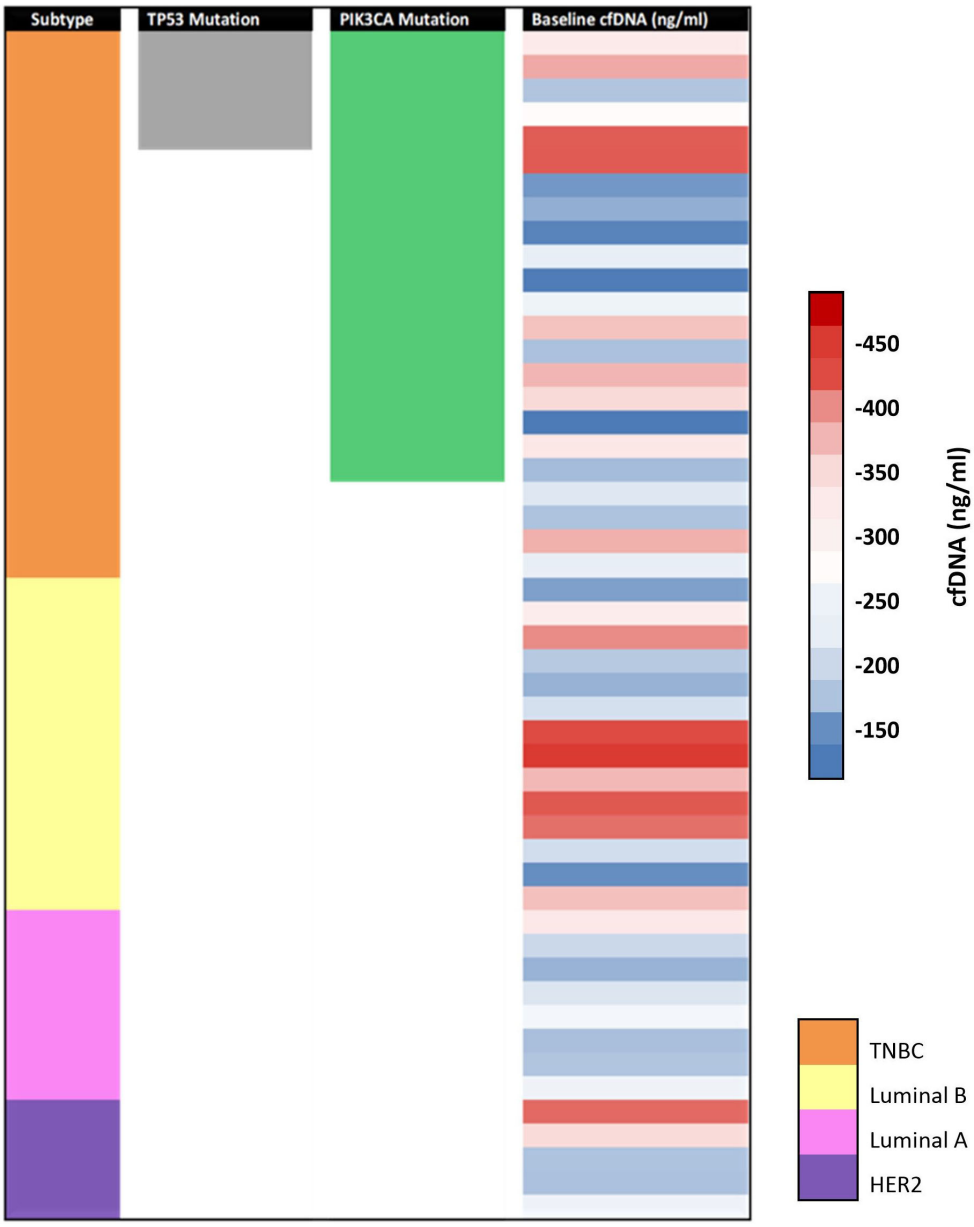
Oncoplot summarizing molecular subtype, mutation status (TP53 and PIK3CA), and cfDNA levels (baseline cfDNA and follow-up cfDNA) across 50 histologically confirmed breast cancer patients.

To provide an integrated overview of the study cohort, an oncoplot (**Figure 2**) was generated, summarizing molecular subtypes, mutation status (TP53 and PIK3CA), and cfDNA levels at baseline and follow-up.

### 3.2 Baseline cfDNA levels in cases vs. controls

Baseline cfDNA levels were assessed in all breast cancer patients (cases) and 20 age-matched healthy female controls. The mean baseline cfDNA level in cases was 316.86 ± 175.57 ng/mL, significantly higher than controls (84.73 ± 31.64 ng/mL). The median baseline cfDNA concentration [IQR] among cases was 297.00 [148.00–404.00] ng/mL, compared to 77.25 [56.12–107.62] ng/mL in controls. Mann–Whitney U test confirmed a significant difference (U = 960.00, p < 0.001).

**Figure 3:**
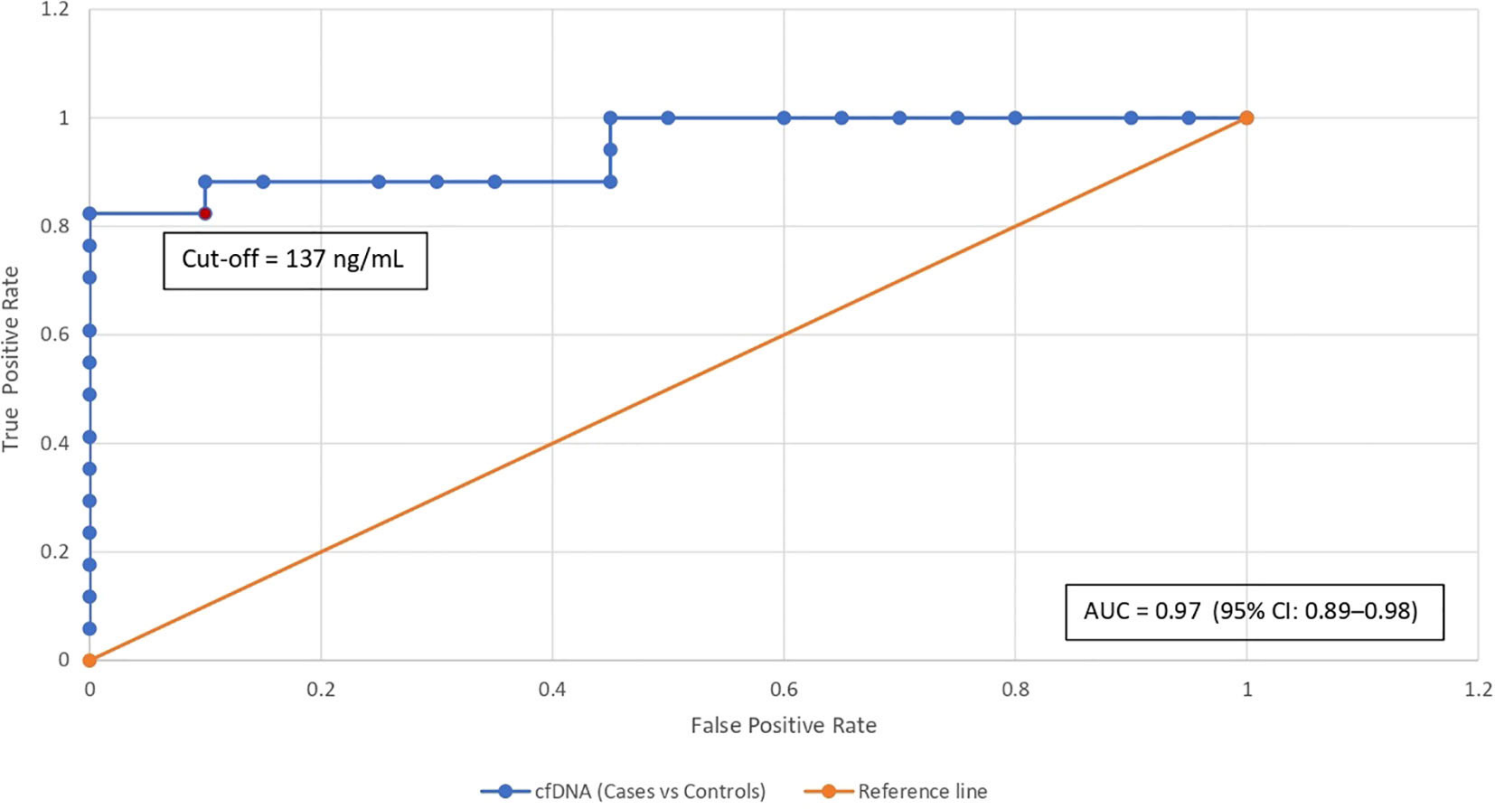
ROC curve for cfDNA concentration as a diagnostic marker. The curve plots sensitivity (true positive rate) against 1−specificity (false positive rate) across varying cfDNA thresholds, yielding an area under the curve (AUC) of 0.97 (95% CI: 0.89–0.98). The red dot marks the optimal threshold of 137.0 ng/mL, at which the true positive rate was 0.7647 and the false positive rate was 0.15.

Receiver Operating Characteristic (ROC) curve analysis (**Figure 3**) yielded an Area Under the Curve (AUC) of 0.97 (95% CI: 0.89–0.98), indicating excellent diagnostic performance. Using the Youden Index, the optimal cut-off value of 137.0 ng/mL was identified, achieving a sensitivity of 91% and specificity of 92%, with a True Positive Rate (TPR) of 0.7647 and a False Positive Rate (FPR) of 0.15. At the 137.0 ng/mL cfDNA threshold, the test achieved a sensitivity of 91%, specificity of 92%, positive predictive value (PPV) of 96%, and negative predictive value (NPV) of 82%.

**Figure 4:**
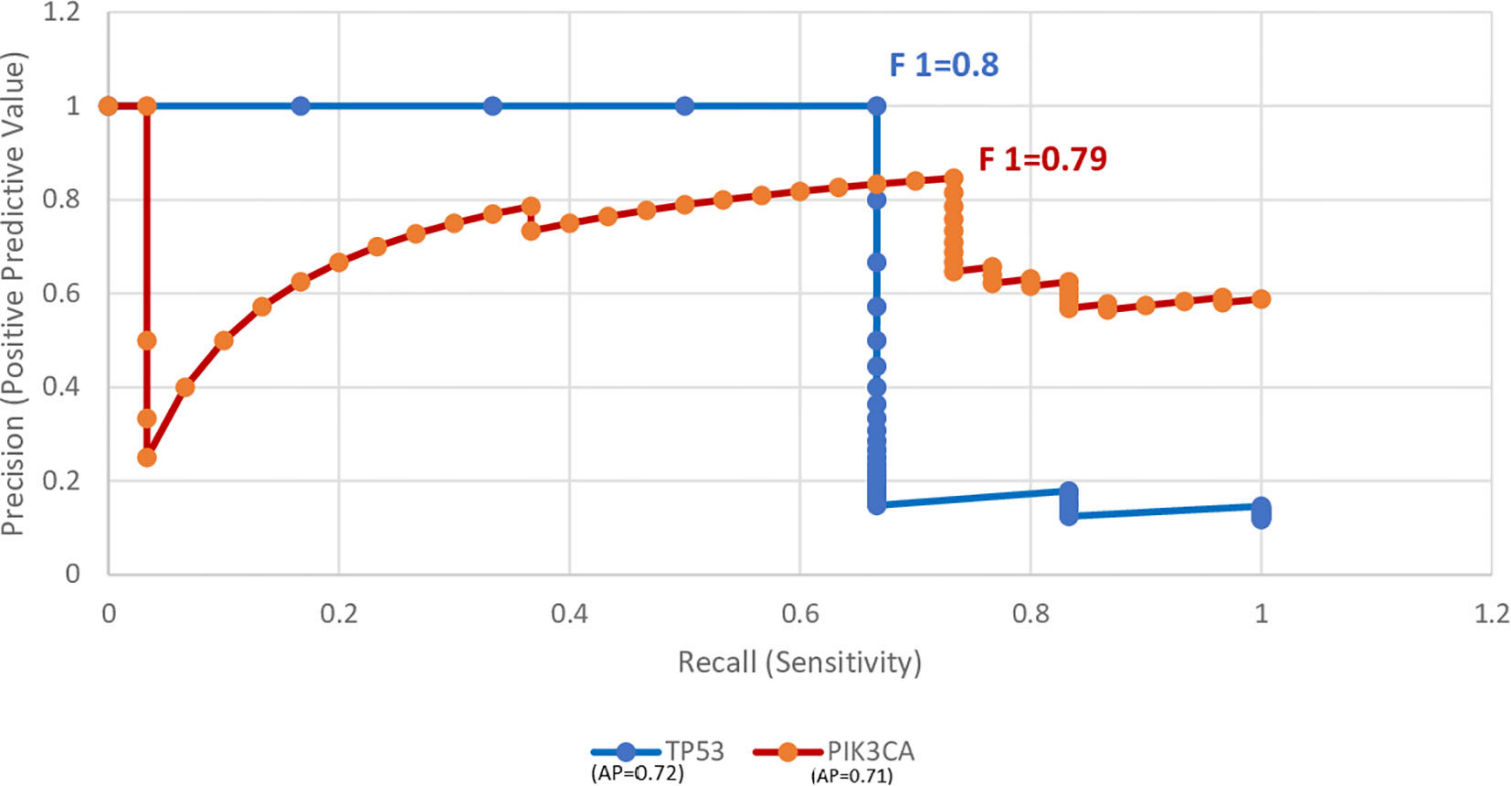
Precision–recall curve overlay for baseline cfDNA in predicting TP53 and PIK3CA mutations. Curves represent the trade-off between sensitivity and precision across thresholds.

Precision–Recall (PR) curve analysis (**Figure 4**) evaluated cfDNA’s ability to distinguish mutation-positive cases (TP53 and PIK3CA). The average precision (AP) was 0.72 for TP53 and 0.71 for PIK3CA. Maximum F1 scores were 0.80 for TP53 (precision = 1.00, recall = 0.67) and 0.79 for PIK3CA (precision = 0.85, recall = 0.73), demonstrating good predictive capability.

### 3.3 cfDNA in relation to tumor burden and biology

**Figure 5:**
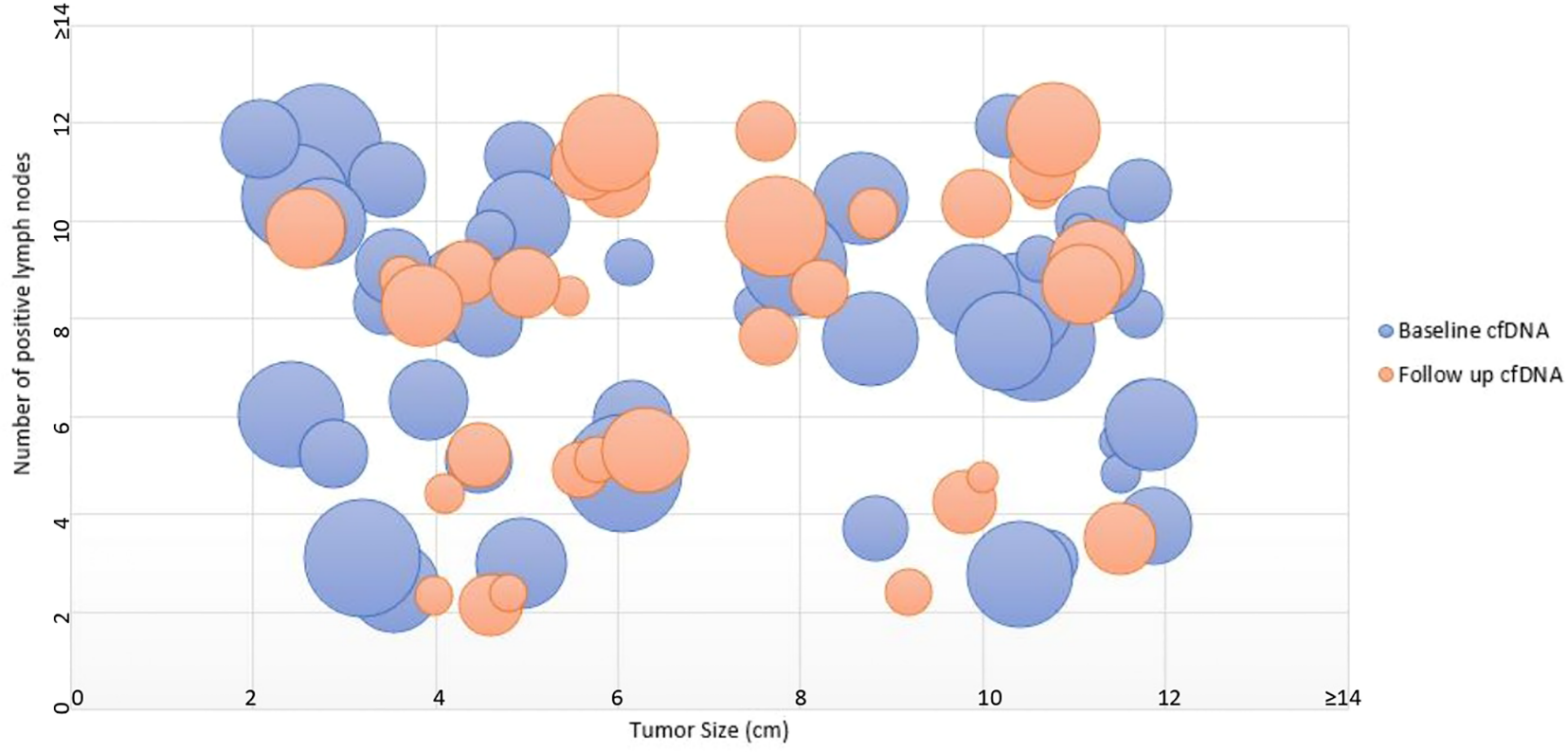
Bubble plot illustrating baseline and follow-up cfDNA according to tumor size and nodal burden. Each bubble represents one patient. The X-axis indicates tumor size in centimeters, and the Y-axis indicates the number of positive lymph nodes. Tumor size and nodal involvement are shown as absolute observed values within the study cohort. Bubble size reflects cfDNA concentration, with baseline and follow-up measurements distinguished by color.

**Figure 5** illustrates a quadrant-based bubble plot depicting baseline cfDNA levels in relation to tumor size (cm) on the X-axis and the number of positive lymph nodes on the Y-axis, both plotted as absolute observed values. At baseline, patients with tumor size ≥5 cm and lymph node involvement ≥5 (n = 11) had a median cfDNA level of 516.0 ng/mL, with 6 cases exceeding 500 ng/mL. In contrast, patients with tumor size <5 cm and lymph node involvement <5 (n = 8) demonstrated a lower median cfDNA level of 291.5 ng/mL, with only 2 cases above 500 ng/mL. Overall, the plot visually reflects increasing cfDNA levels with greater tumor burden.

Baseline cfDNA levels varied significantly across molecular subtypes (Kruskal–Wallis test, p = 0.0018). TNBC had the highest median concentration (367.0 ng/mL), followed by HER2-enriched (345.0 ng/mL), Luminal B (278.0 ng/mL), and Luminal A (125.0 ng/mL). Among non-responders, TNBC (overall ΔcfDNA −420.0 ng/mL; TP53-mutated subset 517.0 ng/mL) and HER2-enriched (407.0 ng/mL) maintained higher cfDNA levels than Luminal A (185.0 ng/mL) and Luminal B (312.0 ng/mL).

Group-level analysis of ΔcfDNA (follow-up – baseline) showed the highest increase in non-responders with TP53-mutated TNBC (+229.0 ng/mL, n = 3), followed by wild-type TNBC (+150.5 ng/mL, n = 15) and HER2-enriched tumors (+62.0 ng/mL, n = 5). Luminal A and Luminal B subtypes had more modest increases (+60.0 ng/mL and +34.1 ng/mL, respectively).

### 3.4 cfDNA and mutation status (TP53, PIK3CA)

Comparative analysis showed a significant association between TP53 mutations and elevated cfDNA levels. At baseline, TP53-mutated cases had a median cfDNA level of 350.0 ng/mL versus 119.0 ng/mL in wild-type cases (U = 36.0, p = 0.0039; Cliff’s Delta = −0.72). At follow-up, similar differences were noted (324.5 ng/mL vs. 181.0 ng/mL, U = 4.0, p = 0.0033; Cliff’s Delta = −0.74). cfDNA levels in TP53 wild-type cases ranged from 72.0 to 225.0 ng/mL, partially overlapping with the upper limit observed in normal controls (107.62 ng/mL). In contrast, TP53-mutated cases exhibited markedly higher cfDNA levels ranging from 295.0 to 398.0 ng/mL, with no overlap, indicating a distinct cfDNA elevation pattern. Baseline cfDNA levels were higher in TP53-mutated TNBC (410.0 ng/mL) compared to wild-type TP53 in Luminal A (105.0 ng/mL; p = 0.161), although not statistically significant. The observed overlap in cfDNA levels between TP53 wild-type tumors and controls may contribute to diagnostic uncertainty in borderline cases, underscoring the importance of interpreting cfDNA values in conjunction with clinical and molecular parameters.

**Figure 6:**
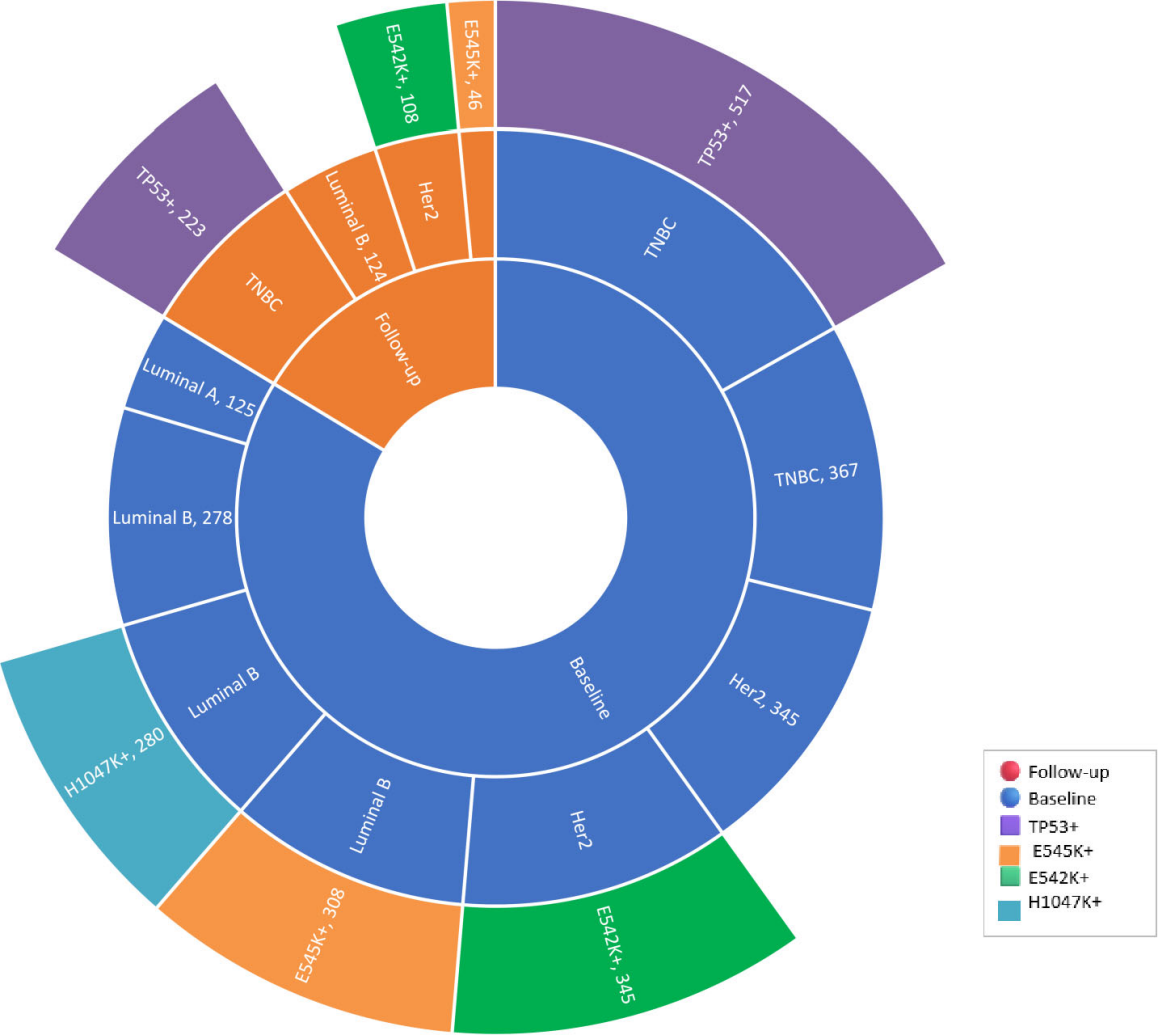
Sunburst plot illustrating the hierarchical distribution of cfDNA levels in breast cancer patients according to molecular subtype and mutation status, at baseline and follow-up timepoints.

In contrast, PIK3CA-mutated cases showed no significant difference in cfDNA levels compared to wild-type at baseline (300.0 ng/mL vs. 297.0 ng/mL; p = 0.7688) or follow-up (p = 0.7178). A hierarchical sunburst plot (**Figure 6**) mapped cfDNA distribution across timepoints, molecular subtypes, and mutation status, illustrating layered variation.

### 3.5 Longitudinal changes in cfDNA and treatment response

Among the 50 patients, 32 had paired cfDNA samples available for baseline and follow-up analysis. The mean change in cfDNA was −66.27 ng/mL (range: −250.0 to +361.0 ng/mL), with a significant overall decrease observed post-treatment (W = 127.0, p = 0.030; effect size, r = 0.384). After therapy, cfDNA levels decreased in 23 patients (72%), increased in 7 (22%), and remained unchanged in 2 (6%).

Clinical response was assessed based on institutional protocols using a combination of physical examination and available imaging (ultrasound, mammography, or CT, as applicable), although standardized RECIST criteria were not uniformly applied. Non-responders, as identified by this combined clinical-radiological assessment, predominantly exhibited either an increase or no significant change in cfDNA levels.

**Figure 7:**
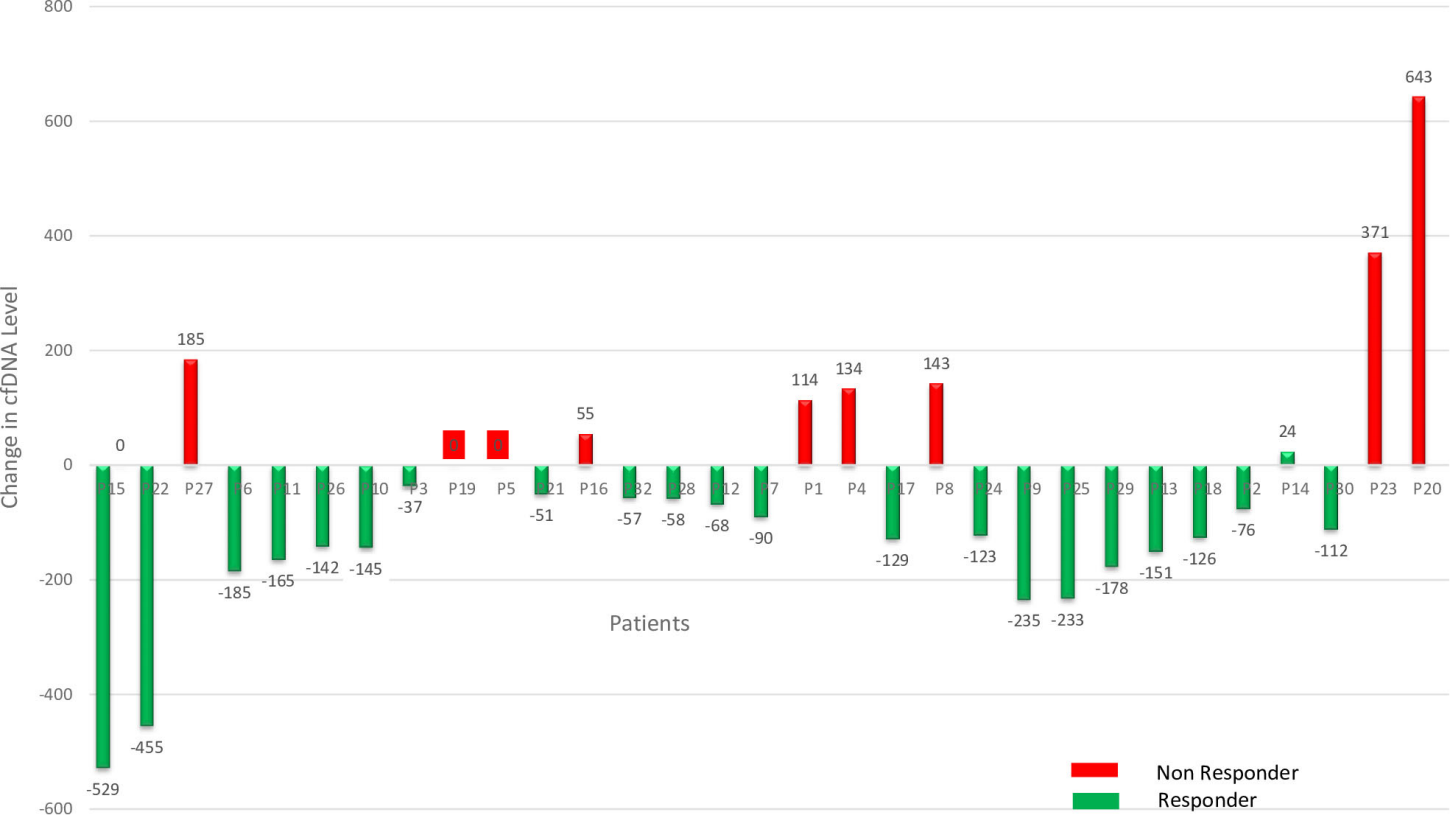
Waterfall plot illustrating the change in cfDNA levels (Post-treatment – Pre-treatment) across all 50 patients. Bars are color-coded by treatment response status: green indicates clinical responders, and red indicates non-responders. A negative ΔcfDNA indicates a reduction in circulating tumor DNA post-therapy.

A waterfall plot (**Figure 7**) illustrates the distribution of ΔcfDNA across the cohort. The median absolute cfDNA reduction was greater among responders (128.5 ng/mL) compared to non-responders (64.5 ng/mL), though the difference was not statistically significant (p = 0.284, Mann–Whitney U test; Cliff’s Delta = 0.23).

Subtype-specific trends were also observed. In Luminal B tumors (n = 8), five cases harbored PIK3CA mutations—four of which were responders—while among the three wild-type cases, two responded and one did not. In TNBC, three patients had TP53 mutations, of whom two were non-responders. Among the nine TP53 wild-type TNBC cases, eight showed treatment response. However, comparative evaluation across treatment modalities was not performed and warrants further investigation.

**Figure 8:**
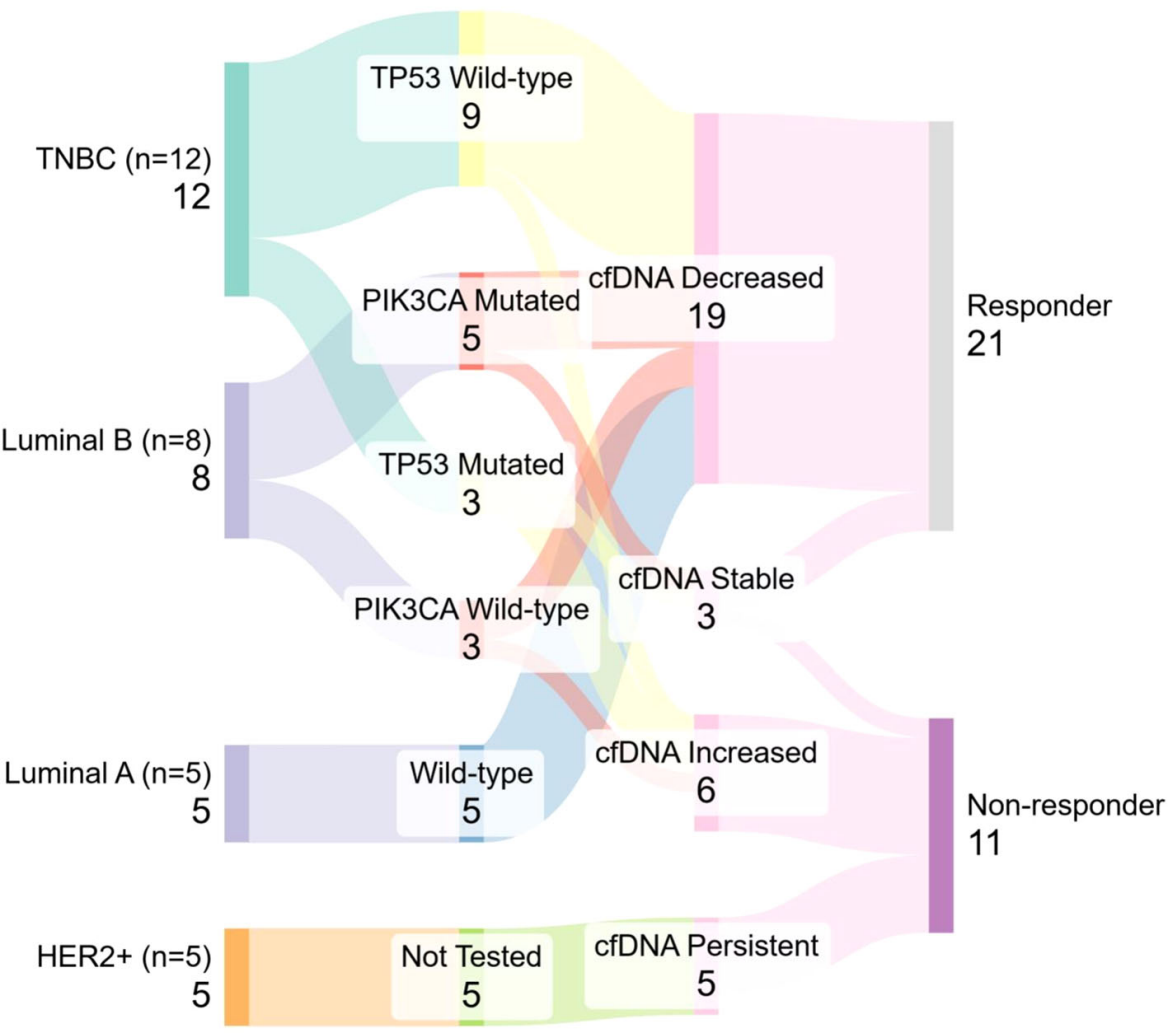
Sankey diagram illustrates the integrative flow from breast cancer molecular subtypes (left) to mutation status, cfDNA dynamics, and treatment response (right).

A Sankey diagram (**Figure 8**) showing the distribution of patients (n = 32) across molecular subtype, mutation status, cfDNA kinetics, and treatment response. Node width is proportional to patient count. Most Luminal A and B patients demonstrated cfDNA decline and clinical response, while TP53-mutated TNBC and HER2-enriched patients tended toward cfDNA persistence or increase and poor response. This integrative view supports subtype- and mutation-informed cfDNA interpretation for treatment monitoring.

## 4 Discussion

cfDNA is a promising non-invasive biomarker for cancer diagnosis, prognosis, and treatment monitoring. This study evaluated cfDNA levels in breast cancer patients, integrating molecular subtypes (TP53, PIK3CA) with baseline and follow-up quantification.

### 4.1 Diagnostic potential of cfDNA in breast cancer detection

cfDNA demonstrated its potential as a robust biomarker for capturing both static and dynamic features of breast cancer. Baseline cfDNA levels were markedly elevated in patients compared to controls, reflecting tumor-derived apoptosis and necrosis. This finding aligns with Karathanasis et al. (17), who observed increased cfDNA levels across all major subtypes, particularly in TNBC and HER2-enriched tumors. ROC analysis showed a high AUC of 0.97, with a threshold of 137.0 ng/mL achieving 91% sensitivity and 92% specificity. These results support cfDNA’s utility as a complementary, non-invasive biomarker for molecular profiling and risk stratification in diagnosed breast cancer patients.

In our cohort, baseline cfDNA levels were highest in TNBC and HER2-enriched subtypes, followed by Luminal B, with Luminal A showing the lowest concentrations. These results are consistent with Pushpanjali et al. (9), who also reported elevated cfDNA in TNBC compared to ER-positive and HER2-positive subtypes (p < 0.001). This distribution reflects the aggressive nature and high proliferation rates of TNBC and HER2-enriched cancers, which are marked by significant genomic instability. These subtype-specific distributions were further visualized using an oncoplot (**Figure 2**), highlighting the variations in cfDNA levels across different molecular categories.

### 4.2 cfDNA correlates of tumor aggressiveness: burden and subtype

The clinicopathological profile highlighted significant tumor burden, with nearly one-third of cases ≥5 cm and over 90% >2 cm, many with lymph node involvement ≥5. This is consistent with studies, including Wang et al. (18), correlating cfDNA with tumor size, stage, and nodal involvement. The quadrant-based bubble plot (**Figure 5**) visualized this association, highlighting how higher cfDNA levels corresponded with greater tumor burden and nodal involvement, reinforcing its value as a surrogate marker for disease extent.

Follow-up analysis indicated that patients occupying high cfDNA quadrants consistently showed evidence of persistent disease, while those with lower cfDNA concentrations were predominantly associated with tumor regression, reinforcing the role of cfDNA in monitoring disease dynamics over time.

In our cohort, TNBC (46%) and Luminal B (28%) were the predominant molecular subtypes, followed by Luminal A (16%) and HER2-enriched (10%). This distribution contrasts with Western datasets, where Luminal A subtypes typically predominate (30–50%) and TNBC and Luminal B together account for only 10–20% of cases (19, 20). In contrast, Indian studies have reported a relatively higher TNBC prevalence of 25–31% (21–23), consistent with our findings and indicative of a distinct regional molecular profile. Contributing factors may include younger age, higher parity, and obesity, common in South Asian populations. The high TNBC proportion in our cohort may reflect its association with elevated cfDNA levels, characteristic of aggressive tumors. This predominance is likely influenced by the tertiary care setting, which manages advanced or atypical cases prioritized for cfDNA and molecular testing. Conversely, Luminal A was less represented, possibly due to minimal cfDNA shedding or limited clinical indications for analysis. Our findings reinforce the regional relevance of TNBC and HER2-enriched subtypes, emphasizing the need for population-specific biomarker validation. Our study provides detailed quantitative stratification of cfDNA across subtypes at baseline, with TNBC (367 ng/mL) and HER2-enriched (345 ng/mL) showing the highest median levels, followed by Luminal B (278 ng/mL) and Luminal A (125 ng/mL). This data underscores cfDNA’s potential for subtype classification, particularly where tissue biopsy is limited, and strengthens its role as a triage or adjunct diagnostic tool in real-world settings.

### 4.3 Mutation profile of TP53 and PIK3CA: population and assay-level insights

In our study, TP53 mutations were identified in 10% of breast cancer cases through cfDNA analysis. Triple-negative breast cancer (TNBC), which typically harbors TP53 mutations in up to 80% of cases (20), represented 30% of our cohort. This proportion is consistent with previous reports (30–35%) in large tissue-based datasets such as TCGA (24). However, the relatively lower mutation frequency observed in our cfDNA-based analysis may reflect technical and biological factors, including the use of an assay-based qPCR approach that may miss very low-frequency.

Our approach prioritized cfDNA quantification rather than targeted mutation enrichment, which may have further contributed to underestimation of TP53 mutational burden. While cfDNA demonstrated subtype- and mutation-associated trends in our cohort, its role in early detection was not assessed in this study. Evaluating its screening potential would require a distinct prospective design involving asymptomatic populations.

PIK3CA mutations were detected in 36% of cases, aligning with global reports of 20–40%, particularly in hormone receptor–positive tumors (19). Typically, the H1047R mutation is the most frequent PIK3CA hotspot, accounting for 35% of all PIK3CA mutations in datasets like TCGA (24) and METABRIC (25), as well as in studies by Martínez-Sáez et al. (26). However, in our cohort, H1047R was found in only 2% of cases, while E545K and E542K were more prevalent at 28% and 6%, respectively. This shift may reflect cohort composition and cfDNA detection sensitivity, as helical domain mutations (E545K, E542K) are more typical of TNBC and Luminal B subtypes.

Cohort composition likely influenced mutation patterns, with TNBC and Luminal B subtypes enriched for helical domain mutations (E545K, E542K), while H1047R predominated in Luminal A, which constituted only 20% of our cohort.

cfDNA detection limitations, including fragment size, allele burden, and amplification efficiency, may reduce detection sensitivity for low-frequency mutations like H1047R. Ethnic and geographic variations could also explain these discrepancies, as mutation landscapes in Indian populations differ from Western cohorts and are often underrepresented in global datasets. These observations underscore the importance of population-specific genomic profiling and the careful interpretation of cfDNA results, considering both biological variability and technical limitations.

These findings emphasize the importance of interpreting cfDNA-based mutation data within regional molecular contexts and assay-specific limitations. While PIK3CA mutation rates align with global data, the shift in hotspot mutations suggests potential population-specific genomic traits. In contrast, the underrepresentation of TP53 mutations highlights the need for more sensitive, mutation-targeted cfDNA approaches to improve clinical relevance in aggressive subtypes.

The association between mutation status and cfDNA levels was evaluated at baseline and follow-up. TP53-mutated cases consistently showed higher cfDNA concentrations than their wild-type counterparts. Specifically, median baseline cfDNA levels were 410.0 ng/mL for TP53-mutated TNBC and 125.0 ng/mL for Luminal A with wild-type TP53, though this difference was not statistically significant (p = 0.161). While not significant, this trend aligns with the established link between TP53 mutations, genomic instability, and increased cfDNA release in aggressive subtypes. These findings suggest that mutation-driven biological behavior may influence cfDNA dynamics, warranting larger studies for confirmation. Although TP53-mutated cases showed consistently higher cfDNA levels compared to wild-type, the limited number of such cases (n = 5) constrains statistical power. These findings, while suggestive, should be interpreted cautiously and validated in larger, mutation-enriched cohorts.

Conversely, PIK3CA-mutated tumors exhibited no significant difference in cfDNA levels compared to wild-type cases at either timepoint (p > 0.7). This is consistent with their predominance in Luminal A and B subtypes, known for lower proliferation rates and reduced cfDNA shedding. These observations match prior reports, including Chen et al. (27), indicating that PIK3CA mutations impact treatment resistance more than cfDNA quantity, suggesting a functional rather than quantitative influence.

The regional genomic patterns observed in this cohort underscore the potential utility of cfDNA as a pragmatic, minimally invasive approach for mutation assessment and disease monitoring in routine clinical practice. In resource-constrained settings, such assay-based cfDNA testing may serve as a triage tool to identify patients who warrant more comprehensive genomic profiling, enabling rational use of advanced sequencing resources. Additionally, the mutation spectrum identified in this population may inform the development of population-tailored cfDNA assays applicable to similar healthcare settings.

### 4.4 Predictive modeling and utility in mutation detection

We assessed whether baseline cfDNA levels could predict mutation positivity using Precision–Recall modeling (**Figure 3**). TP53 and PIK3CA showed moderate predictive performance, with average precision (AP) scores of 0.72 and 0.71, and F1 scores of 0.80 and 0.79, respectively. While elevated cfDNA levels may correlate with mutation presence, their specificity for reliably identifying mutations was limited.

Although cfDNA quantification reflects tumor burden and biology, it remains insufficient for precise mutation detection. Notably, persistently elevated cfDNA post-treatment in TP53-mutated cases suggests potential for tracking residual molecular disease. Enhanced detection will require integration of mutation-specific methods like droplet digital PCR (ddPCR) or next-generation sequencing (NGS), especially within precision oncology frameworks.

### 4.5 cfDNA dynamics post-treatment and correlation with response

Serial cfDNA analysis highlighted heterogeneity in treatment response, reinforcing ΔcfDNA as a dynamic biomarker. While many responders showed marked cfDNA decline, some non-responders had only modest reductions, indicating biological variability. Persistently elevated or rising cfDNA levels post-treatment were more frequent in non-responders, suggesting residual disease.

Prior studies support the prognostic value of cfDNA dynamics in breast cancer. Magbanua et al. (28) showed that persistent cfDNA elevation post-neoadjuvant therapy predicted poor response and relapse, especially in aggressive subtypes. Jongbloed et al. (29) linked cfDNA decline during treatment to improved progression-free survival in metastatic cases. Similarly, Hassan et al. (30) reported that elevated postoperative cfDNA independently predicted recurrence in early-stage disease.

In our cohort, mutation-specific trends showed differing cfDNA dynamics between responders and non-responders. TP53-mutated TNBC cases showed poor response, with two of three classified as non-responders and displaying persistently elevated cfDNA. In contrast, most TP53 wild-type or unassessed TNBC cases responded to therapy, suggesting a more favorable phenotype. However, the lack of a fixed follow-up interval across patients limits direct comparison of serial cfDNA changes. Although a median follow-up of 6 months was maintained, inter-patient variability (3–9 months) could affect cfDNA dynamics.

Among non-responders (n = 9), TP53-mutated TNBC showed the highest mean post-treatment cfDNA increase (+229.0 ng/mL), followed by TP53 wild-type TNBC (+150.5 ng/mL) and HER2-enriched subtypes (+62.0 ng/mL). In contrast, indolent phenotypes like Luminal A and B showed smaller increases (+60.0 ng/mL and +34.1 ng/mL, respectively), likely reflecting differences in tumor aggressiveness, residual burden, and cfDNA shedding kinetics. While treatment regimens varied, a trend of more pronounced cfDNA decline was observed in patients receiving neoadjuvant chemotherapy, suggesting that cfDNA kinetics may be influenced by the type and timing of treatment. Further controlled studies are warranted to confirm this observation.

These findings align with prior studies and underscore the clinical utility of cfDNA monitoring. Combining cfDNA trends, mutation profiles, and molecular subtypes provides a nuanced view of tumor dynamics. Persistent cfDNA elevation, especially in TP53-mutated TNBC and HER2-enriched cases may indicate residual disease or early relapse, warranting intensified surveillance.

PIK3CA mutations, mainly seen in Luminal B cases, align with their known enrichment in hormone receptor–positive tumors, particularly Luminal A and B [Reinhardt et al. (31)]. Most PIK3CA-mutated Luminal B patients responded to treatment, consistent with their intermediate aggressiveness and partial treatment sensitivity. Overall, these results support incorporating longitudinal cfDNA tracking into follow-up, especially for high-risk or therapy-resistant subtypes (32).

This study offers several novel contributions. It is among the first from India to perform dual timepoint cfDNA analysis stratified by molecular subtype and mutation status, capturing real-time tumor dynamics. The use of Precision–Recall curve analysis on baseline cfDNA adds a predictive layer, assessing its ability to enrich for TP53 and PIK3CA mutations, an approach rarely reported in cfDNA studies. We also observed a distinct PIK3CA mutation profile, with high E545K and low H1047R frequency, contributing valuable regional genomic data. Establishing a cfDNA threshold of 137 ng/mL with 91% sensitivity and 92% specificity supports its translational use in biopsy-limited contexts, highlighting its real-world applicability. TP53 mutations were linked to persistently elevated cfDNA and poor response, while PIK3CA mutations mainly in Luminal B, correlated with more favorable cfDNA trends, underscoring the utility of mutation-integrated cfDNA profiling for personalized treatment.

However, this study has certain limitations. The single-center design and small subgroup sizes may limit statistical power and generalizability. Follow-up cfDNA samples were collected at variable intervals, which could influence the interpretation of ΔcfDNA trends. Mutation analysis was based solely on cfDNA without matched tumor tissue and employed quantitative rather than mutation-specific assays, potentially underestimating low-frequency variants. The sample may also overrepresent aggressive subtypes due to referral patterns at a tertiary care center. Additionally, treatment regimens were not standardized across patients, which may have affected cfDNA dynamics. To enhance clinical correlation, future studies should incorporate treatment-homogeneous cohorts, standardized imaging-based response assessments, and parallel tissue mutation profiling.

## 5 Conclusion

Our findings support cfDNA as a versatile biomarker in breast cancer diagnostics and management. Elevated baseline cfDNA in aggressive subtypes (TNBC, HER2-enriched) and persistently high or rising levels post-treatment suggest its potential role in risk stratification and residual disease monitoring. ΔcfDNA trends offer additional prognostic insight, while Precision–Recall analysis highlights cfDNA’s potential to enrich for mutation-positive cases. The high prevalence of PIK3CA E545K emphasizes the importance of population-specific validation. As a minimally invasive, real-time indicator of tumor dynamics, cfDNA holds strong potential to complement tissue-based diagnostics and support precision oncology, particularly for longitudinal monitoring and in resource-constrained settings. With further validation, cfDNA-guided strategies could transform breast cancer care.

## Data Availability

The original contributions presented in the study are included in the article/**Supplementary Material**. Further inquiries can be directed to the corresponding author/s.
