# Supplementary material for: Circulating cell-free DNA as an integrative biomarker in breast cancer: correlation with molecular subtypes, mutation status, and treatment response
Source: Front Oncol. 2026 Feb 18;16:1737210. doi: 10.3389/fonc.2026.1737210 (PMC12956535; doi:10.3389/fonc.2026.1737210)
Supplement: Supplementary file 1 [file Table1.docx]

**Supplementary Table S1-** Commercial TaqMan castPCR™ mutation detection assays used for TP53 and PIK3CA mutation analysis.

| Gene | Target exon(s) / mutation(s) | Assay technology | Manufacturer | Description |
| --- | --- | --- | --- | --- |
| PIK3CA | Exon 9 (E542K, E545K) and Exon 20 (H1047R/H1047L) | TaqMan Mutation Detection Assays (castPCR™) | Applied Biosystems, Thermo Fisher Scientific | Hotspot mutation assays targeting the most frequently reported activating PIK3CA mutations in breast cancer |
| TP53 | Exons 5–8 (hotspot regions) | TaqMan Mutation Detection Assays (castPCR™) | Applied Biosystems, Thermo Fisher Scientific | Mutation screening assays covering commonly mutated TP53 exons |
